# Case Report: Fibromatosis-like metaplastic breast cancer: a case with detection of three genetic alterations, accompanied by local recurrence, lymph node metastasis, and distant metastasis

**DOI:** 10.3389/fonc.2026.1865144

**Published:** 2026-08-21

**Authors:** Xin Zhang, Xiaoqing Cheng, Yue Shi, Yufan Xu

**Affiliations:** Department of Pathology, Sir Run Run Shaw Hospital, Zhejiang University School of Medicine, Hangzhou, China

**Keywords:** breast cancer, fibromatosis-like metaplastic carcinoma, metaplastic carcinoma, molecular pathology, recurrence

## Abstract

Fibromatosis-like metaplastic carcinoma (FLMC) is defined as a rare histologic subtype of metaplastic carcinoma within the 2019 World Health Organization (WHO) classification framework for breast tumors. We present a case of FLMC demonstrating a notably atypical clinical presentation. A 73-year-old woman presented with a self-detected breast mass. Radiological and pathological evaluations both indicated potential malignancy. Histological examination showed a lesion composed predominantly of spindle cells and displaying an infiltrative growth pattern. Immunohistochemical staining confirmed co-expression of cytokeratin and p63 within the spindle cell component, supporting a diagnosis of FLMC. During follow-up, the patient developed local recurrence along with lymph node and distant metastases, leading to subsequent next-generation sequencing (NGS) to investigate underlying molecular alterations. Through NGS, we identified a deletion mutation in *PIK3R1*, a frameshift mutation in *RUNX1*, and *MYC* gene amplification. These findings provide preliminary molecular clues for the relatively aggressive biological behavior of FLMC.

## 1 Introduction

Metaplastic carcinoma represents a histologically heterogeneous subset of invasive breast carcinomas, defined by the divergent differentiation of neoplastic epithelial cells toward squamous and/or mesenchymal lineages (1). According to histological classification, metaplastic carcinomas can be categorized into the following subtypes: spindle cell carcinoma, mixed metaplastic carcinoma, low-grade adenosquamous carcinoma (LGASC), metaplastic carcinoma with heterologous mesenchymal differentiation, squamous cell carcinoma, and fibromatosis-like metaplastic carcinoma (FLMC). Fibromatosis-like metaplastic carcinoma is an exceedingly uncommon form of triple-negative breast cancer, constituting less than 1% of all invasive breast malignancies (2). Due to its bland spindle cell morphology, it is often misdiagnosed as a benign mesenchymal proliferative lesion or tumor, making the diagnosis potentially challenging from a morphological perspective (2–4). Therefore, a systematic integration of morphological, immunohistochemical, and molecular profiling is essential to establish a definitive diagnosis and to prevent inappropriate clinical management. Compared to other subtypes of metaplastic carcinomas, FLMC generally exhibits a relatively favorable clinical prognosis. In certain cases, local recurrence may be observed, whereas regional lymph node involvement and distant metastasis are infrequently encountered (3, 5). Therefore, ensuring clear surgical margins is critical for achieving local disease control and improving long-term outcomes.

Although the distinctive morphological and immunophenotypic characteristics of FLMC have been well-documented in the literature, the molecular pathogenesis remains to be fully elucidated. Previous studies have identified several recurrent genetic alterations in this rare breast cancer entity. Webersinke et al. reported that TERT promoter mutations represent one of the most characteristic genetic alterations in FLMC (6). According to investigations by Zhong (7) and colleagues, fibromatosis-like metaplastic carcinoma (FLMC) exhibits substantially increased mutation rates in both the *PIK3CA* gene and *TERT* promoter regions, suggesting the potential significance of these genetic alterations in the pathogenesis and progression of this malignancy. In addition, Takano et al. observed that two out of three cases of fibromatosis-like spindle cell carcinomas (FLSCCs) harbored 9p21.3 deletions and confirmed that the copy number variations (CNVs) in FLSCCs do not overlap with other metaplastic breast carcinomas (MBCs), showing relatively few molecular-level alterations (8).

We herein report a case of FLMC exhibiting a constellation of relatively uncommon clinical manifestations, including local recurrence, axillary lymph node involvement, and distant organ metastasis. NGS was performed to investigate the underlying molecular basis of its clinical presentation.

## 2 Case presentation

### 2.1 Clinical presentation

A self-detected, painless small nodule in the left breast was the presenting complaint in a 73-year-old postmenopausal woman with a history of more than half a month. Breast MRI demonstrated an oval-shaped nodule (24 × 18 mm) in the left breast’s upper outer quadrant, characterized by lobulated and spiculated margins with heterogeneous internal enhancement (BI-RADS 4B). No calcifications were detected. Multiple lymph nodes were observed in the left axilla, demonstrating a prominent morphology with preserved corticomedullary architecture. The patient had no history of other neoplasms or surgeries, except for a uterine myomectomy performed over two decades ago. Subsequently, a segmental resection of the affected breast was undertaken at our institution for the current condition.

### 2.2 Pathology and immunohistochemistry

**Figure 1:**
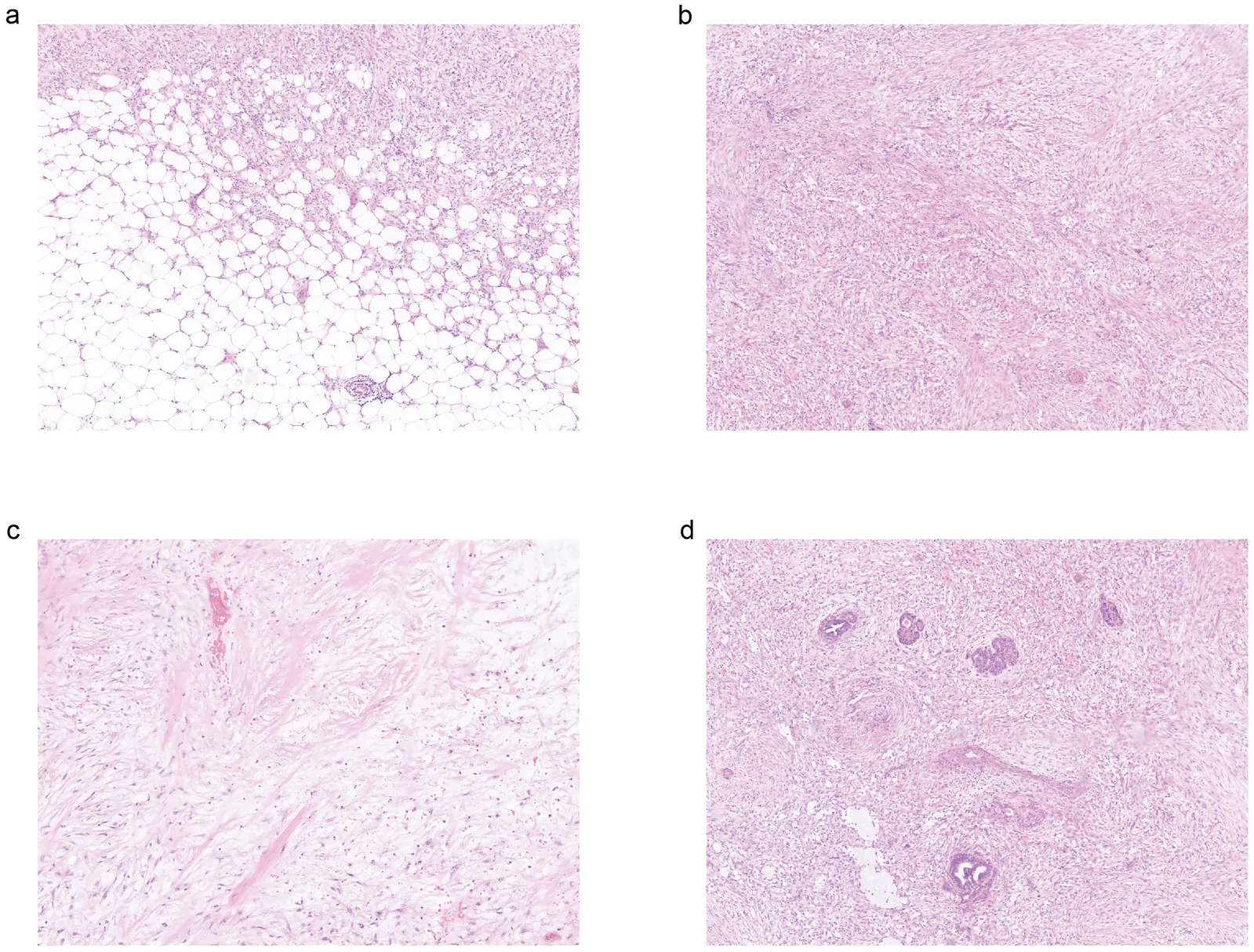
**(a)** Low-power view shows the relationship between the mass and the surrounding fibroadipose tissue. **(b)** Microscopic examination revealed spindle cells arranged in fascicles. **(c)** Hyalinized collagen bundles and amorphous myxoid matrix among spindle cells. **(d)** Normal-appearing ducts surrounded by spindle cells with scattered lymphocytic infiltration.

Grossly, the tumor was unencapsulated with poorly defined margins and exhibited a gray-white, solid cut surface. Pathological examination of the intraoperative frozen section showed that the tumor exhibited invasive growth with mild cellular atypia, diagnosed as spindle cell tumor. Subsequently, routine histopathological examination was performed on the specimen, demonstrating a 2.8 × 2 × 1.6 cm nodule that was completely excised with a 6-mm free margin. On the H&E-stained slides, the tumor exhibited an irregular infiltrative growth pattern into the surrounding adipose tissue (**Figure 1a**). It was arranged in a fascicular, matted pattern, predominantly composed of spindle cells with bland morphology and pale-staining cytoplasm (**Figure 1b**). A subset of tumor cells exhibited visible nucleoli, and mitoses were extremely rare. Focally, hyalinized collagen bands were interspersed among the spindle cells, and amorphous myxoid matrix was noted in some areas (**Figure 1c**). Entrapped normal ducts and scattered inflammatory cell infiltration were observed within the tumor (**Figure 1d**). The tumor was composed almost exclusively of spindle cells, with only rare, scattered glandular elements identified in localized areas. Importantly, no overt squamous differentiation, heterologous mesenchymal components, or epithelioid tumor cell nests were observed.

Based on these morphological findings, the differential diagnosis encompassed pseudoangiomatous stromal hyperplasia (PASH), fibromatosis, inflammatory myofibroblastic tumor (IMT), nodular fasciitis (NF), and FLMC. Therefore, we performed an extensive immunohistochemical panel to establish a definitive diagnosis.

**Figure 2:**
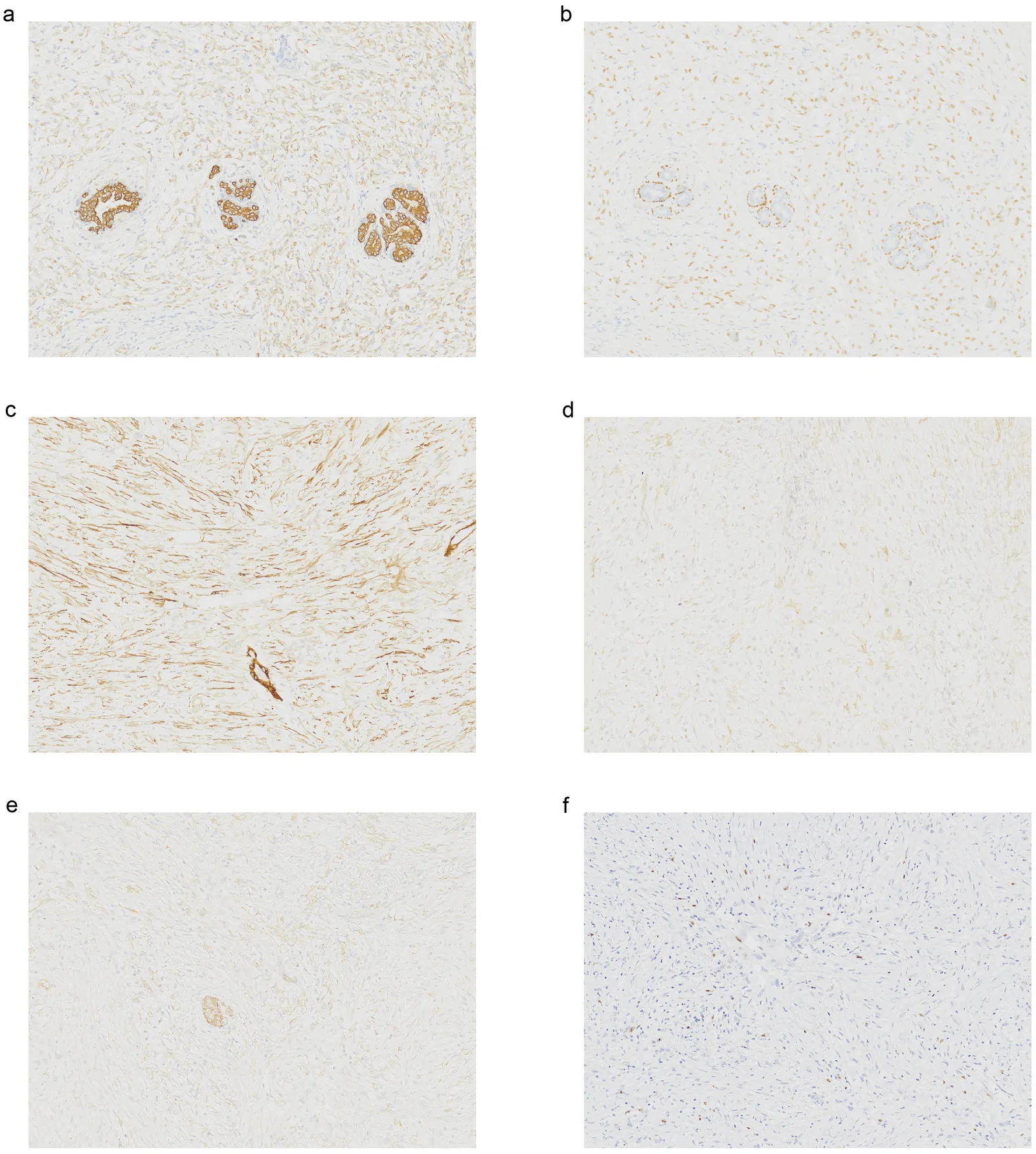
Staining patterns of CK-pan **(a)**, p63 **(b)**, actin **(c)**, SMA **(d)**, β-catenin **(e)**, and Ki-67 **(f)**.

On immunohistochemical (IHC) staining, the spindle cells diffusely expressed pancytokeratin (CK-pan) (**Figure 2a**), CK5/6, CK7, p63 (**Figure 2b**), and actin (**Figure 2c**), while smooth muscle actin (SMA) exhibited weak expression (**Figure 2d**). Some normal mammary ducts served as positive internal controls for CK-pan. Furthermore, this case exhibited a triple-negative immunophenotype, characterized by the absence of human epidermal growth factor receptor 2 (HER2), progesterone receptor (PR), and estrogen receptor (ER) expression, while nuclear β-catenin (**Figure 2e**) expression was concomitantly negative. The Ki-67 (**Figure 2f**) immunostaining demonstrated a proliferative index of approximately 10%.

The diagnoses of IMT, NF, and PASH were excluded based on the following findings: absent expression of ALK, CD34, and CD10, and absence of nuclear β-catenin expression; a triple-negative ER/PR/HER2 profile; co-expression of CK-pan and p63; and spindle cell morphology with focal epithelioid features and low mitotic activity. These findings collectively established the diagnosis of FLMC.

### 2.3 Molecular findings

Next-generation sequencing (NGS) and copy-number variation (CNV) analysis were performed to characterize the genetic alterations. Genomic DNA extracted from formalin-fixed paraffin-embedded (FFPE) tissue was subjected to targeted sequencing using a commercial 168-gene panel (Burning Rock Biotech, Guangzhou, China), encompassing 268.5 kb of the human genome covering 68 lung cancer-related genes and 100 additional cancer-associated genes. Libraries were constructed with sheared DNA fragments (200–400 bp) following end-repair, adapter ligation, and hybridization-based capture. Paired-end sequencing (2 × 150 bp) was performed on the Illumina NextSeq 500 platform, with a mean target depth exceeding 1,000× for tissue samples.

Raw reads were aligned to the human reference genome (hg19) using BWA-MEM v.0.7.10. Duplicate marking and local realignment were performed with GATK v.3.2, and variant calling for single nucleotide variations (SNVs) and small insertions/deletions (indels) was executed using VarScan v.2.4.3. Variants with a supporting read depth <100× were filtered out; SNVs and indels required a minimum of 8 and 5 supporting reads, respectively. Germline polymorphisms with a population frequency >0.1% in public databases (ExAC, 1000 Genomes, dbSNP, and ESP6500) were excluded. Remaining somatic variants were annotated via ANNOVAR.

For CNV analysis, read-depth coverage per captured interval was normalized against GC-bias and sequencing noise, using a reference baseline established from more than 50 non-CNV control samples. The limit of detection for copy number (CN) gain is CN > 2.25 for hotspot genes (such as EGFR, TP53, ERBB2, and MET) and CN > 2.5 for others, while for CN loss is CN < 1.75 for hotspot genes and CN < 1.5 for others. Putative pathogenicity of the identified variants was interpreted using a combination of population frequency filters, in silico prediction tools (SIFT, PolyPhen-2), and curated databases (ClinVar, COSMIC), following the ACMG/AMP guidelines for somatic variants. Chromosomal translocations were analyzed using Factera v.1.4.3.

**Figure 3:**
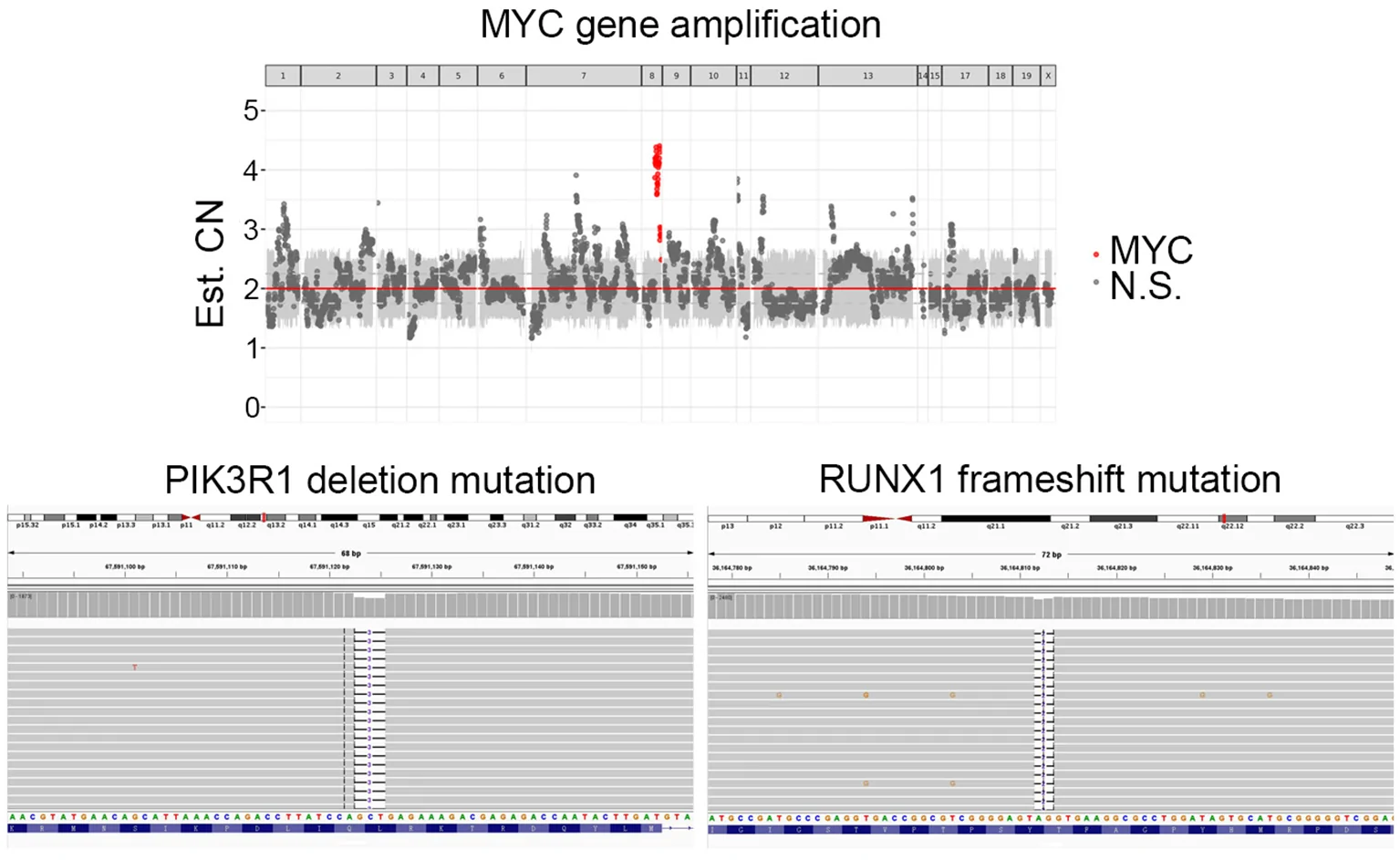
NGS genetic analysis reveals three pathogenic mutations in *PIK3R1*, *RUNX1*, and *MYC*.

Genetic analysis of the primary tumor identified three pathogenic variants (**Supplementary Table 1**): a *PIK3R1* deletion mutation (exon 13 p.L573del), a *RUNX1* frameshift mutation (exon 9 p.Y355fs), and *MYC* gene amplification. Notably, the same *PIK3R1* deletion mutation (exon 13 p.L573del) was identified in both primary and relapsed cases (**Figure 3**).

### 2.4 Follow-up and clinical course

The patient’s clinical course was marked by sequential disease progression despite initial complete resection. Local recurrence in the left breast occurred 20 months after the initial surgery, accompanied by ipsilateral axillary lymph node metastases. Both the recurrent and metastatic lesions histologically resembled the primary tumor. A subsequent 3-cm pulmonary metastatic lesion was detected 32 months post-surgery. After biopsy confirmation, the patient underwent chemoradiotherapy. The detailed chronological sequence of interventions, margin status, and clinical outcomes is summarized in **Table 1**.

**Table 1:** Chronological summary of clinical course, interventions, and outcomes.

Time point (months)	Event	Intervention	Margin/pathology status	Outcome/imaging finding
0	Initial diagnosis (index surgery)	Segmental resection of left breast	6 mm free margin; tumor size 2.8cm; FLMC confirmed	Complete resection achieved
20	Local recurrence + ipsilateral axillary lymph node metastasis	Re-excision + lymph node sampling	Histologically similar to primary tumor; LN positive	Radiotherapy
32	Distant metastasis (lung)	Core biopsy + palliative thoracic radiotherapy	Metastatic FLMC confirmed (histologically similar)	3 cm pulmonary lesion; CRT
40	Died of disease	Palliative/best supportive care	N/A	Died approximately 8 months after distant metastasis (at 40 months post-surgery)

FLMC, fibromatosis-like metaplastic carcinoma; LN, lymph node; N/A, not applicable; CRT, chemoradiotherapy.

The patient ultimately died of the disease at 40 months after the initial surgery, approximately 8 months after the detection of pulmonary metastasis.

## 3 Discussion

The breast spindle cell lesions encompass a diverse diagnostic category. This spectrum includes entities ranging from reactive and benign proliferations to a variety of malignant neoplasms (9–11). Histologically, these lesions often show overlapping features, making their diagnosis particularly challenging in some instances, especially on intraoperative frozen section examination. Therefore, a comprehensive understanding of the morphological, immunohistochemical, and molecular characteristics is crucial for differential diagnosis, accurate diagnosis and appropriate therapeutic intervention (12).

However, in the evaluation of the broad spectrum of spindle cell proliferative lesions, fibromatosis should be considered the primary differential diagnosis. Histologically, the lesion is characterized by proliferating spindle-shaped fibroblasts and myofibroblasts arranged in intersecting fascicles, which can form distinctive finger-like projections that infiltrate the surrounding breast tissue. Importantly, epithelial cell clusters are absent. Immunohistochemically, the spindle cells are negative for CK-pan and p63 but positive nuclear staining for β-catenin. Secondly, inflammatory myofibroblastic tumor of the breast is also one of the lesions requiring differentiation. Currently, reports on this specific breast lesion remain relatively scarce. Histologically, it is defined by a proliferation of spindle cells intermingled with a dense inflammatory infiltrate, which may efface the normal mammary lobular architecture. Immunophenotypically, the neoplastic spindle cells commonly express SMA, vimentin, and desmin, while ALK expression is observed in approximately half of the cases (13). In contrast, nodular fasciitis in the mammary region most commonly develops within the subcutaneous tissue and rarely infiltrates the glandular parenchyma. The lesion typically demonstrates rapid progression and presents with distinct margins. Histologic examination reveals proliferating spindle fibroblasts and myofibroblasts embedded in a loose, culture-like stromal matrix. The stroma can undergo hyaline or myxoid transformation, and extravasation of red blood cells is commonly noted. In addition, it is necessary to differentiate pseudoagiomatous stromal hyperplasia (PASH) of the breast. This benign condition is characterized by a fibromyoblastomatous proliferation within the extralobular nonspecific stroma, associated with prominent keloid-like fibrous hyperplasia. The stromal component typically contains intricate, interconnecting pseudovascular slit-like spaces. Immunohistochemically, the spindle cells that line these spaces show positive staining for CD34 and vimentin, while remaining negative for CK-pan. A recently published study identified five cases of FLMC that histologically mimicked PASH, emphasizing the importance of CK-pan and CD34 immunostaining to distinguish malignancy from benign hyperplasia (14). Mammary fibromatosis should be included among the lesions considered during differential diagnosis (15). Histologically, it manifests as long, sweeping bundles of spindle cells containing oval to elongated nuclei and exhibiting minimal mitotic activity. A hallmark finding is the presence of gently curved, thin-walled blood vessels. These vessels are frequently more noticeable under low-power magnification, a feature attributable to mild perivascular edema and the comparatively lower density of neoplastic spindle cells around the vessels versus the endothelial cell nuclei. From an immunohistochemical standpoint, around 70 to 80 percent of these cases show abnormal nuclear positivity for β-catenin.

Regarding cellular origins, spindle cell lesions of the breast demonstrate remarkable heterogeneity involving multiple lineages. These lesions may arise from either myoepithelial cell proliferation or mammary stromal cell expansion (16). Additionally, epithelial-mesenchymal transition (EMT) facilitates spindle cell metaplasia within neoplastic epithelial cells, thereby culminating in the acquisition of a mesenchymal cell-mimicking phenotype. Occasionally, breast carcinomas may completely lose their epithelial morphology and exhibit pure spindle cell morphology. Lamovec (17) et al. reported an aggressive FLMC case with four local recurrences, axillary and pulmonary metastases, and biphasic differentiation with progressive epithelial enrichment during recurrence, suggesting that epithelial component proportion may correlate with metastatic risk.

Our case predominantly exhibited a nodular growth pattern with focal infiltrative features (18). The tumor was composed of fibroblastic spindle cells exhibiting minimal to no significant cytological atypia, accompanied by occasional glandular elements in localized regions. These features provide critical diagnostic clues for differential diagnosis. In immunohistochemical assessment, the initial approach requires demonstration of epithelial derivation through CK-pan, CK-low, and CK5/6. The detection of epithelial antigen expression within the spindle cell compartment serves as a hallmark diagnostic feature for metaplastic breast carcinoma. Ultrastructural analysis offers definitive proof regarding the epithelial derivation of these spindle cells (19).

From a molecular perspective, recent studies demonstrate that MBCs harbor significantly higher mutational frequencies in *PI3K/AKT/mTOR* pathway-related genes compared to other breast tumors (20). Metaplastic breast carcinomas demonstrate significantly higher frequencies of *PIK3CA*,

*PIK3R1*, and *TERT* gene mutations compared to triple-negative breast carcinomas. Among MBC subtypes, *TERT* promoter mutations show a particularly prominent association with spindle cell variant MBC, representing the second most frequent mutation after *TP53* gene mutation. Notably, spindle cell and other low-grade metaplastic breast carcinomas exhibit significantly reduced *TP53* mutation rates compared to high-grade variants (33, 34). In our case, next-generation sequencing identified mutations in *PIK3R1*, *RUNX1*, and *MYC* genes, with no detectable *TP53* mutations-a finding consistent with the low-grade histological features typically associated with this tumor subtype. To our knowledge, while these genetic alterations have been well characterized in triple-negative breast cancers (TNBCs) and metastatic breast carcinomas in large cohort studies, this is the first report to document their coexistence in a single case of FLMC with aggressive clinical progression. Nevertheless, given that this observation derives from a single case and lacks functional validation, we emphasize that these findings should be interpreted as hypothesis-generating rather than conclusive. Specifically, *MYC* amplification is frequently observed in metastatic breast carcinomas exhibiting squamous and spindle cell differentiation (35), and *PIK3R1* mutations are among the common driver events in the *PI3K/AKT/mTOR* pathway in metaplastic carcinomas. Given the absence of functional experiments or comparative genomic analyses in this single case, we cannot establish a causal relationship between these molecular alterations and the aggressive phenotype. Instead, we propose these findings as a hypothesis-generating observation-namely, that the concurrent *PIK3R1/RUNX1* mutation and *MYC* amplification may have potentially contributed to the unusual metastatic progression in this patient. However, whether these alterations are true drivers or merely concurrent bystanders remains to be elucidated through larger cohort studies and functional investigations.

FLMC is conceptualized as a distinct low-grade morphological variant within the spectrum of spindle cell metaplastic carcinomas. Its relatively indolent histological appearance has led some authors to propose the term “fibromatosis-like metaplastic tumor.” However, the documented risks of local recurrence (16 cases) and distant metastasis (6 cases) in the English literature over the past 30 years justify its continued classification as a carcinoma (**Table 2**). A key insight from this literature review is that metastatic potential appears to correlate with primary tumor size and the proportion of epithelial components, while local recurrence risk is primarily determined by the completeness of surgical excision (17). These observations have important clinical implications: they support a management strategy that prioritizes achieving clear surgical margins to minimize local recurrence, while avoiding overly aggressive systemic therapy given the low incidence of lymph node metastasis. Nevertheless,

**Table 2:** Summary of research on FLMC of the breast over the past 30 years .

Author (reference)	Case, n	Age/mean (range)	Side	Size/mean (range-cm)	Recurrence (interval range, months)	Surgical margin	Axillary lymph node metastasis	Distant metastasis	Initial treatment	Adjuvant therapy	Follow-up and outcomes (months)
Gobbi et al. (21)	30	63.4[40-80]	15L 9R 6NA	2.7[1.2-7.0]	[5-72] 7 after excision 1 after WE	NA	0	0	5 MxT + LN 5 WE + LN 5 WE 8 excision 7 NA	1 CT 1 RT 1 CT + RT 1 RT + HT	Range 6–88 [18/30] 1 DOD (Died 8 mos after reexcision; cause unknown)
Sneige et al. (4)	24	66[55-85]	17L 7R	2.8[1.0-5.0]	2[5-32] 2 after LE 0 after AT	1 negative in recurrence; 23 NA	0	2; 1 lung 1 lung, inguinal soft tissue and bone	9 MRM 3 MRM + RT 1 MRM + CT 1 LE + LN 6 excision 4 NA	5 RT 1 CT	Range 5–90 [18/24]
Kinkor et al. (22)	4	[54-72]	NA	[2.0-3.5]	NA	NA	NA	2	NA	NA	NA
Rekhi et al., 2007 (23)	1	77	L	2.0	0	Negative	0	0	LE	RT	16
Podetta et al. (24)	2	79[72-85]	1NA 1L	4.4[3.0-5.7]	0	NA	0	0	1 MRM 1 WE + LN	1 RT	Range 21-27
Lamovec et al. (17)	1	48	L	1.0	3[15.3]	Negative	1	Lung and bone	LE	RT + CT	72
Nonnis et al. (12)	1	73	L	2.0	1[9]	Negative (1mm)	0	0	LE	NO	62
Kinkor et al. (25)	1	76	L	4.5	NA	N	NA	NA	LE	NO	NA
Rito et al. (26)	3	66[54-72]	1L 2R	5.0	1	NA	NA	1 lung	1 core biopsy 2 WE	1 CT + RT	Range 21-27[2/3]
Li et al. (27)	3	50.6[47-54]	NA	2.5[2.0-3.5]	1[15]	NA	0	0	1 LE + LN 1 SM 1 MRM	2 RT	Range 7-26
Zhao et al. (3)	3	57[51-65]	2L 1R	3.5[3.0-4.0]	1[13]	NA	0	0	2 MRM 1 WE	1 CT + RT 2 RT	Range 12-49
Victoor et al. (2)	1	65	L	1.9	0	Negative (<1mm)	0	0	LE	NO	24
Takatsuka et al. (28)	1	56	L	1.3	0	Negative	0	0	MRM	CT	44
Pham et al. (5)	1	51	L	2.2	0	NA	0	0	MRM	NO	3
Bao et al. (29)	2	54[52-56]	2R	4.25[4.0-4.5]	0	NA	0	0	2 MRM	NO	Range 11-15
Wang et al. (30)	1	56	R	2.5	0	Negative	0	0	LE	NO	24
Wu et al. (31)	1	58	R	1.5	0	Negative	0	0	MRM	CT	36
Laokulrath et al. (32)	1	53	L	2.3	0	NA	0	0	LE + LN	NO	7

L, left; R: right; NA, not available; MxT, mastectomy; LE, lumpectomy; WE, wide local excision; MRM, modified radical mastectomy; SM, Simple Mastectomy; LN, axillary lymph node dissection; CT, chemotherapy; RT, radiotherapy; HT, hormonal therapy.

the occurrence of rare distant metastases underscores the need for standardized long-term follow-up to enable timely detection of disease progression and accurate prognostic assessment.

When compared with previously reported FLMC cases, our case shares several features with the published literature: the patient’s age (73 years) falls within the reported range (40–85 years), and the tumor size (2.8 cm) is comparable to the mean sizes reported in large series (2.7-2.8 cm) (4, 21). However, our case differs in three clinically relevant aspects. First, the time to local recurrence (20 months) and distant metastasis (32 months) was shorter than the mean interval reported in the literature (range 5–72 months for recurrence, and up to 88 months for metastasis) (21, 26). Second, while lymph node metastasis is documented in only one previous case (17), our patient developed ipsilateral axillary lymph node involvement at the time of local recurrence. Third, the concurrent presence of *PIK3R1*, *RUNX1*, and *MYC* genetic alterations has not been previously reported in FLMC, although these alterations have been individually described in triple-negative breast carcinomas and metaplastic carcinomas. Collectively, these comparisons suggest that our case represents an aggressive end of the FLMC spectrum, and the genetic findings may offer a potential molecular explanation for this unusual clinical behavior-a hypothesis that requires validation in future studies.

## 4 Conclusion

As a rare subtype of metaplastic carcinoma, breast FLMC necessitates consideration of a wide spectrum of differential diagnoses. For such cases, comprehensive tissue sampling is essential to identify focal epithelial differentiation, while definitive diagnosis necessitates multidisciplinary evaluation incorporating imaging findings and immunohistochemical profiling. In our case, the tumor was overwhelmingly composed of bland spindle cells, with rare glandular elements observed locally. Through NGS, we identified three genetic alterations: *PIK3R1* mutation, *RUNX1* mutation, and *MYC* amplification. The clinical course was highly aggressive, with local recurrence, lymph node involvement, and distant metastasis occurring during follow-up. Collectively, these findings provide preliminary data expanding the molecular spectrum of FLMC, while also underscoring that the pathogenetic significance of these concurrent alterations-and the clinical relevance of minor epithelial components-requires further validation in larger series and functional models. This case highlights the importance of both standardized histological reporting and integrated clinicopathological-molecular evaluation for this rare subtype.

## Data Availability

The raw data supporting the conclusions of this article will be made available by the authors, without undue reservation.
