# Supplementary material for: Case Report: Fibromatosis-like metaplastic breast cancer: a case with detection of three genetic alterations, accompanied by local recurrence, lymph node metastasis, and distant metastasis
Source: Front Oncol. 2026 Aug 21;16:1865144. doi: 10.3389/fonc.2026.1865144 (PMC13541470; doi:10.3389/fonc.2026.1865144)
Supplement: Supplementary file 1 [file Table1.docx]

Supplementary-Table1:

The table summarizes the gene mutation profiles of the primary breast tumor and the distant lung metastasis.

**Primary Breast Lesion**

| Sample_ID | Gene | Description | AF | DP | CHR | POS | REF | ALT | Var_Type | Mutation_Type | Exon_Number | HGVSc | HGVSp | HGVSp_Short | HGVS | Transcript_ID |
| --- | --- | --- | --- | --- | --- | --- | --- | --- | --- | --- | --- | --- | --- | --- | --- | --- |
| CQ23000635FFP | RUNX1 | p.Y355fs | 9.55% | 1979 | 21 | 36164811 | TAG | T | INDEL | frameshift_variant | 9 | c.1062_1063del | p.Tyr355fs | p.Y355fs | NM_001754.4(RUNX1):c.1062_1063del(p.Tyr355fs) | NM_001754.4 |
| CQ23000635FFP | PIK3R1 | p.L573del | 21.39% | 1753 | 5 | 67591122 | AGCT | A | INDEL | conservative_inframe_deletion | 13 | c.1717_1719del | p.Leu573del | p.L573del | NM_181523.2(PIK3R1):c.1717_1719del(p.Leu573del) | NM_181523.2 |
| CQ23000635FFP | MYC | cn_amp | 3.5 | NA | 8 | 8q24.21 | 4 | 2 | CNV | cn_amp | NA | cn_amp | NA | NA | cn_amp | NM_002467.4 |

**Distant Lung metastasis**

| Sample_ID | Gene | Description | AF | DP | CHR | POS | REF | ALT | Var_Type | Mutation_Type | Exon_Number | HGVSc | HGVSp | HGVSp_Short | HGVS | Transcript_ID |
| --- | --- | --- | --- | --- | --- | --- | --- | --- | --- | --- | --- | --- | --- | --- | --- | --- |
| CQ23000634FFP | PIK3R1 | p.L573del | 7.54% | 2016 | 5 | 67591122 | AGCT | A | INDEL | conservative_inframe_deletion | 13 | c.1717_1719del | p.Leu573del | p.L573del | NM_181523.2(PIK3R1):c.1717_1719del(p.Leu573del) | NM_181523.2 |
