# Supplementary material for: Case Report: Fibromatosis-like metaplastic breast cancer: a case with detection of three genetic alterations, accompanied by local recurrence, lymph node metastasis, and distant metastasis
Source: Front Oncol. 2026 Aug 21;16:1865144. doi: 10.3389/fonc.2026.1865144 (PMC13541470; doi:10.3389/fonc.2026.1865144)
Supplement: Supplementary file 2 [file Table2.docx]

Table S2. **Differential diagnosis of spindle cell lesions of the breast**

| Lesion | **Morphology** | **IHC profile** | **Common molecular alterations** |
| --- | --- | --- | --- |
| **FLMC** | Bland spindle cells in fascicles, rare glandular elements, infiltrative growth | CK‑pan+, p63+, CK5/6+, actin+, SMA weak+, ER‑/PR‑/HER2‑, β‑catenin‑, Ki‑67 ~10% | PIK3R1*,* PTEN, TERT |
| **IMT** | Spindle cells with prominent lymphoplasmacytic infiltrate, may efface lobular architecture | ALK+ (~50%), SMA+, desmin+, vimentin+, CK‑pan‑ | ALK rearrangements (2p23), ROS1/NTRK3 fusion |
| **Nodular fasciitis** | Rapidly growing, circumscribed, spindle cells in loose myxoid stroma, extravasated RBCs | SMA+, vimentin+, calponin+, CK‑pan‑, ALK‑, CD34- | USP6 rearrangement or MYH9-USP6 fusion |
| **PASH** | Interanastomosing slit‑like pseudovascular spaces, keloid‑like fibrous hyperplasia | CD34+, vimentin+, SMA+, CK‑pan‑, ER+/PR+ (usually) | No consistent genetic alterations |
| **Mammary fibromatosis** | Long sweeping bundles of bland spindle cells, thin‑walled curved vessels, minimal mitoses | β‑catenin (nuclear)+, SMA+, CK‑pan‑, CD34‑ | CTNNB1 mutations (exon 3) in 45-65% |

Abbreviations: FLMC, fibromatosis‑like metaplastic carcinoma; IMT, inflammatory myofibroblastic tumor; NF, nodular fasciitis; PASH, pseudoangiomatous stromal hyperplasia; IHC, immunohistochemistry; CK, cytokeratin; SMA, smooth muscle actin; ER, estrogen receptor; PR, progesterone receptor; HER2, human epidermal growth factor receptor 2; RBC, red blood cells.
